# Association between uric acid and the risk of depressive symptoms in US adults: results from NHANES 2005–2018

**DOI:** 10.1038/s41598-024-74869-5

**Published:** 2024-10-15

**Authors:** Jinhua Wang, Ming Yang, Haiyan Lin, Jiao Wang

**Affiliations:** 1 Cardiovascular Department, The Quzhou Affiliated Hospital of Wenzhou Medical University (Quzhou People’s Hospital), Quzhou, Zhejiang 324000 China; 2 Neurology Department, The Quzhou Affiliated Hospital of Wenzhou Medical University (Quzhou People’s Hospital), Quzhou, Zhejiang 324000 China

**Keywords:** Serum uric acid, Depressive symptoms, NHANES, Weighted logistic regression, Adults, Diseases of the nervous system, Depression

## Abstract

**Background:**

This study explores the relationship between serum uric acid(UA) levels and depression. UA is the final product of purine metabolism in the human body, possessing certain physiological functions such as blood pressure regulation, antioxidation, DNA protection, and anti-aging, thereby drawing attention for its potential role in preventing and treating depression.

**Methods:**

This cross-sectional study includes 32,424 participants aged ≥ 20 years from the National Health and Nutrition Examination Survey (NHANES) conducted between 2005 and 2018, generating a nationally representative database. Depressive symptoms were assessed using the Patient Health Questionnaire-9. Serum uric acid concentration was measured using the uricase-peroxidase coupled method, and participants were divided into quartiles of serum uric acid concentration. Weighted data were calculated according to analysis guidelines. The association between serum uric acid and depressive symptoms was analyzed using weighted multivariable logistic regression models and restricted cubic spline regression analyses. Subgroup analyses were also performed.

**Results:**

Among 32,424 participants, 3,421 were defined as having depressive symptoms. The crude prevalence of depressive symptoms was 10.5% (weighted prevalence: 9.086% [95% confidence interval: 9.032–9.139%]). Compared with the first quartile, individuals with higher UA levels had a decreased risk of depressive symptoms by 9% (OR: 0.910, 95% CI: 0.797–10.40), 14.6% (OR: 0.854, 95% CI: 0.741–0.983), and 20.5% (OR: 7795, 95% CI: 0.680–0.930), respectively. Further restricted cubic spline regression analysis revealed a nonlinear association between UA and depressive symptoms, with an inflection point of 319.72 µmol/L. Subgroup multivariable weighted logistic regression analysis found that the association between UA and the risk of depressive symptoms remained consistent across all subgroups, demonstrating high stability and reliability.

**Conclusion:**

This study emphasizes a significant nonlinear negative correlation between serum uric acid and depressive symptoms. This suggests that proper control of serum uric acid levels may play a role in preventing and treating depression.

**Supplementary Information:**

The online version contains supplementary material available at 10.1038/s41598-024-74869-5.

## Introduction

Presently, research on depression has brought to light its alarming prevalence and projected that by 2030, depression could emerge as a primary driver of the escalating global disease burden^1–3^. The global point prevalence rate of elevated self-reported depressive symptoms from 2001 to 2020 was 34%. Point prevalence for major depressive disorder (MDD) and dysthymia was 8% and 4%, respectively^4^. Particularly concerning is the United States, where depression affects a striking 8.7% of the population, establishing it as the most prevalent mental disorder in the nation^5^. Depression not only profoundly compromises individual quality of life but also inflicts physical impairments, compromising functions such as muscle strength and motor skills^6^. Moreover, depression closely correlates with the onset or exacerbation of severe conditions like cardiovascular diseases^7^, diabetes^8^, and a marked increase in suicide rates^9^. The ramifications of depression extend beyond individual suffering to encompass substantial economic burdens. Indeed, the economic toll attributed to depression has been steadily mounting. Concurrently, the economic burden soared dramatically by 37.9%, reaching a staggering $326.2 billion in 2020^10^. These economic ramifications encompass direct medical expenses, suicide-related costs, and workplace expenditures, with the latter experiencing particularly notable growth. This escalating economic strain underscores the urgent imperative to bolster research on the etiology, prevention, and treatment of depression to effectively manage the condition and mitigate its profound societal impact.

Uric acid, an end product of purine metabolism in the human body, is subject to various influences, including diet, genetics, and renal function^11,12^. In the context of depression, observations suggest notable phenomena concerning changes in uric acid levels. Studies indicate that high plasma levels of uric acid were associated with low risk of depression hospitalization and antidepressant medication use^13^. Possibly linked to aberrant metabolic processes inherent to depression. Furthermore, the accumulation of uric acid might impact the nervous system^14^. Some investigations propose a correlation between high uric acid levels and neurodegenerative diseases, often accompanied by psychological symptoms akin to depression^15–17^. Although direct literature exploring the uric acid-depression relationship remains limited, speculation arises regarding uric acid’s potential to influence depression onset by affecting nervous system function. Moreover, uric acid could contribute to depression pathogenesis by modulating the antioxidant system^18^. Depression often associates with increased oxidative stress^19^, and alterations in uric acid levels, acting as an antioxidant, may disrupt the body’s redox balance^20^. Changes in uric acid levels may alter the effectiveness of the antioxidant system, leading to changes in oxidative stress and the risk of depression. However, the exact association between serum uric acid and depressive symptoms is still controversial, and there is a lack of large-scale clinical studies.

This cross-sectional study aims to examine the potential association between serum uric acid levels and depression. We utilized data from the National Health and Nutrition Examination Survey (NHANES) conducted between 2005 and 2018 to investigate the relationship between adult depression symptoms and serum uric acid concentrations. Through this study, we seek to shed further light on the potential connection between the two variables, providing new scientific evidence for the prevention and treatment of depression.

## Methods

### Study population

**Figure 1:**
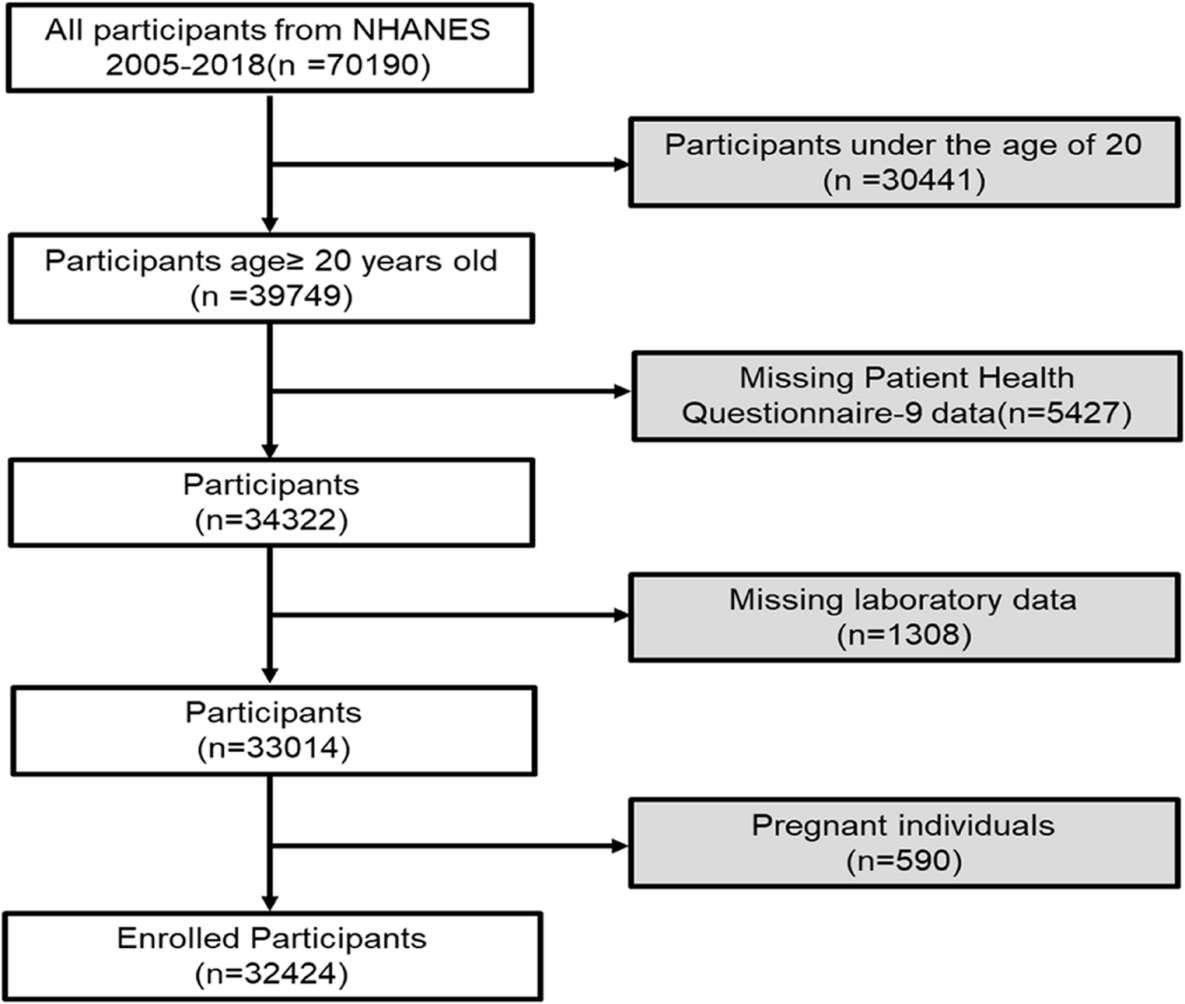
Flowchart of the participant selection from NHANES 2005–2018. NHANES, National Health and Nutrition Examination Survey.

This cross-sectional investigation encompassed participants from the nationally representative continuous cycles of the National Health and Nutrition Examination Survey (NHANES) spanning from 2005 to 2018. The selection of these specific cycles was predicated upon a crucial criterion: all participants in these cycles had undergone assessment through the Patient Health Questionnaire-9 (PHQ-9), regarded as a vital tool for assessing mental health status, particularly depression. From the NHANES 2005–2018 dataset, initially, a total of 70,190 participants were included. Individuals under the age of 20 (*n* = 30,441), those with incomplete PHQ-9 data (*n* = 5,427), individuals with missing laboratory data (*n* = 1,308), and pregnant individuals (*n* = 590) were excluded. Ultimately, 32,424 participants were enrolled in this study (Fig. 1). The total data volume for all continuous variables was 940,325, with 9,526 instances of missing data, which were addressed through multiple imputation. Approval for NHANES was obtained from the ethical review board of the National Center for Health Statistics to ensure adherence to ethical standards and guidelines. Additionally, each participant in the study voluntarily provided written informed consent, underscoring a commitment to ethical research practices and the protection of participant rights and confidentiality.

### Definitions of outcome variables

In this study, we employed the Patient Health Questionnaire-9 (PHQ-9) as a diagnostic tool for assessing depression, which is a self-report instrument based on the Diagnostic and Statistical Manual of Mental Disorders Fourth Edition (DSM-IV). The PHQ-9 comprehensively assesses nine major symptoms and signs associated with depression. Participants were asked to retrospectively review their experiences over the past two weeks and respond on a 4-point scale for each symptom category. Subsequently, we aggregated the scores for each symptom for each participant to derive a total score, ranging from 0 to 27. Consistent with common practice in prior studies^21–23^, depression in this study was defined by setting a threshold of PHQ-9 total score ≥ 10. This specific threshold is widely accepted in clinical and epidemiological research for distinguishing between the presence and absence of depression and has been clinically validated with a high sensitivity and specificity of 88%^24,25^. We strictly adhered to this standardized scoring and diagnostic approach, which not only enhances the reliability and comparability of our study results with existing literature but also further strengthens the robustness of the study findings.

### Definitions of the exposure variable

Serum uric acid concentrations were measured using the Roche Cobas 6000 chemistry analyzer (Roche Diagnostics Corporation, Indianapolis). Serum or plasma samples were processed, and the separated serum or plasma should be extracted from cells within 1 h post-collection. Serum or plasma remains stable for 3 days at 20–25 °C, 7 days at 4–8 °C, 6 months at -20 °C, and longer at -70 °C. Prior to analysis, specimens must be brought to room temperature. The measurement principle involves the oxidation of uric acid by uricase, followed by the action of peroxidase on the peroxide produced from this reaction in the presence of 4-aminophenazone, resulting in a measurable colored product. The assay utilizes a two-point, endpoint reaction, with measurement conducted at 546 nm (secondary wavelength 700 nm).For detailed serum uric acid testing and quality control procedures, please refer to the “Laboratory Methods” section in the NHANES documentation.

### Covariates

Covariates were selected based on existing literature^26,27^ to assess potential confounding factors in the relationship between uric acid and depressive symptoms. These covariates included gender, age, ethnicity, education level, marital status, family poverty income ratio(PIR), smoking status, and alcohol use. Current smoking status was categorized as either smoking or non-smoking, assessed by the question, “Do you currently smoke?” Alcohol use status was similarly categorized as either drinking or non-drinking, assessed by the question, “Do you currently drink alcohol?” Body mass index (BMI) was calculated by trained health technicians at the Mobile Examination Center (MEC) using the formula: weight (kilograms) divided by the square of height (meters). Chronic diseases were assessed through self-reported medical history, including hypertension, diabetes, congestive heart failure, coronary heart disease, stroke, lung disease (emphysema and chronic bronchitis), liver disease, thyroid disease, and cancer. Laboratory covariates were selected based on existing literature^28–30^ and included white blood cell count, lymphocyte count, red blood cell count, hemoglobin, hematocrit, red cell distribution width, platelet count, albumin, alanine aminotransferase, aspartate aminotransferase, blood urea nitrogen, total cholesterol, creatinine, glucose, triglycerides, uric acid, and high-density lipoprotein cholesterol. These covariates were included to control for potential confounding effects and ensure the robustness of the study findings.

### Statistics

All interview and Mobile Examination Center (MEC) examination weights utilized in this study are accessible in the demographic files. Detailed data can be accessed via the following website: https://wwwn.cdc.gov/nchs/nhanes/tutorials/module3.aspx. As MEC examinations constituted a subset of the surveyed interviewees, we amalgamated the MEC examination weights for analysis. The NHANES surveys conducted between 2005 and 2018 encompassed seven survey cycles spanning 14 years. Weighting of the data was performed according to guidance provided by NCHS analysts on combining multiple cycles and constructing appropriate weights.

To investigate the variation in variable characteristics across different uric acid levels, we initially categorized continuous uric acid variables into four groups using quartiles. For continuously distributed variables within each group, we employed weighted ANOVA to assess differences in their means. Conversely, for non-normally distributed continuous variables, we utilized the weighted Kruskal-Wallis H test for analysis. Similarly, for categorical variables, we used weighted chi-square tests or Fisher’s exact tests to evaluate differences among four groups. Prior to ANOVA, we rigorously conducted tests for normality and homogeneity of variance to ensure the accuracy of our analytical results. Furthermore, we stratified participants into two groups based on the presence of depressive symptoms and compared baseline variables between the groups using weighted t-tests, weighted chi-square tests, or Fisher’s exact tests, as appropriate. For skewed continuous variables, we employed the weighted Wilcoxon rank-sum test to compare between-group differences, aiming to comprehensively elucidate the relationship between depressive symptoms and various variables.

To further explore the potential association between serum uric acid and depressive symptoms, we constructed three distinct logistic regression models. Model 1 served as the baseline model without adjustments, while Model 2 adjusted for age and sex relative to Model 1. Model 3 underwent more comprehensive adjustments, encompassing age, sex, ethnicity, education level, marital status, smoking, alcohol use, hypertension, diabetes, congestive heart failure, coronary heart disease(coronary heart disease, angina pectoris, and heart attack), stroke, lung diseases (emphysema, chronic bronchitis), liver disease, thyroid disease, cancer, family poverty income ratio, body mass index, waist circumference, white blood cell count, lymphocyte count, red cell count, hemoglobin, hematocrit, red cell distribution width, platelet count, albumin, alanine aminotransferase, aspartate aminotransferase, blood urea nitrogen, total cholesterol, creatinine, glucose, triglycerides, and high-density lipoprotein cholesterol.

In sensitivity analyses, we utilized restricted cubic spline analysis (RCS) with three knots to evaluate potential nonlinear associations between serum uric acid and depressive symptoms, adjusting for covariates consistent with Model 3. Additionally, we employed two-piecewise logistic regression models to examine the relationship between uric acid levels and depressive symptoms, including potential inflection points.

To gain further insights into the relationship between uric acid and depressive symptoms among different subgroups, we conducted subgroup analyses by stratifying participants based on age, gender, ethnicity, education level, body mass index, marital status, smoking, alcohol use, diabetes, coronary heart disease, congestive heart failure, hypertension, and serum creatinine levels. Interaction terms were included to assess heterogeneity between subgroups, allowing for a more flexible exploration of potential nonlinear patterns.

All statistical analyses were conducted using R version 4.2.1 from the R Foundation for Statistical Computing in Vienna, Austria (http://www.r-project.org). Statistical significance was set at a two-tailed *P*-value of < 0.05 to ensure the robustness and reliability of the study findings.

## Results

### Characteristics

Among 32,424 participants, 3,421 were defined with depressive symptoms. The crude prevalence of depressive symptoms was 10.5%(weighted prevalence: 9.086%[95%CI:9.032–9.139%]). The participants in the first quartile of UA had the highest incidence of depressive symptoms (11.72%).Demographic and relevant clinical characteristics are summarized in Table 1. The mean age of all participants was 50.18 years, with 50.1% being female. A considerable proportion of participants were Non-Hispanic White (43.37%), and 52.62% had reached a college education level. Additionally, 59.61% reported being married or living with a partner. In contrast to the lowest quartile of UA, participants in the highest quartile were older, had a higher proportion of males, were more likely to be Non-Hispanic White, had a higher likelihood of having a High School or Equivalent education level, and were more likely to be married or living with a partner The prevalence of Smoking, Alcohol use, Hypertension, Diabetes, Congestive heart failure, Coronary heart disease, Stroke, Liver disease, and Cancer gradually increased with rising uric acid levels. Furthermore, Body mass index, Waist Circumference, WBC, RBC, Hemoglobin, Hematocrit, Blood urea nitrogen, Creatinine, Glucose, and Triglycerides all increased gradually as the uric acid quartile increased.

**Table 1:** Weighted baseline characteristics of participants.

Variables	Overall	Q1	Q2	Q3	Q4	*P*
	32424	*n* = 8822	*n* = 8051	*n* = 7860	*n* = 7691	
Age (yr)	50.18(17.69)	47.42(17.01)	49.77(17.73)	50.96(17.70)	52.95(17.91)	< 0.001
Gender(%)						< 0.001
Male	16178(49.90)	1862(21.11)	3578(44.44)	4983(63.40)	5755(74.83)	
Female	16246(50.10)	6960(78.89)	4473(55.56)	2877(36.60)	1936(25.17)	
Ethnicity(%)						< 0.001
Mexican American	5072(15.64)	1601(18.15)	1350(16.77)	1213(15.43)	908(11.81)	
Other Ethnicity	6508(20.07)	1924(21.81)	1616(20.07)	1533(19.50)	1435(18.66)	
Non-Hispanic White	14,062(43.37)	3653(41.41)	3491(43.36)	3474(44.20)	3444(44.78)	
Non-Hispanic Black	6782(20.92)	1644(18.64)	1594(19.80)	1640(20.87)	1904(24.76)	
Education level (%)						< 0.001
Less Than 11th Grade	7881(24.31)	2105(23.86)	2013(25.00)	1892(24.07)	1871(24.33)	
High School or Equivalent	7483(23.08)	1848(20.95)	1868(23.20)	1869(23.78)	1898(24.68)	
College or AA degree	9622(29.68)	2664(30.20)	2321(28.83)	2334(29.69)	2303(29.94)	
College or above	7438(22.94)	2205(24.99)	1849(22.97)	1765(22.46)	1619(21.05)	
Marital Status(%)						0.016
Married/Living with partner	19327(59.61)	5222(59.19)	4688(58.23)	4727(60.14)	4690(60.98)	
Widowed/Divorced/Separated	7335(22.62)	2005(22.73)	1852(23.00)	1752(22.29)	1726(22.44)	
Never married	5762(17.77)	1595(18.08)	1511(18.77)	1381(17.57)	1275(16.58)	
Smoking (%)						< 0.001
Yes	14726(45.42)	3441(39.00)	3533(43.88)	3807(48.44)	3945(51.29)	
No	17698(54.58)	5381(61.00)	4518(56.12)	4053(51.56)	3746(48.71)	
Alcohol use (%)						< 0.001
Yes	23763(73.29)	6130(69.49)	5923(73.57)	5894(74.99)	5816(75.62)	
No	8661(26.71)	2692(30.51)	2128(26.43)	1966(25.01)	1875(24.38)	
Hypertension(%)						< 0.001
Yes	11908(36.73)	2281(25.86)	2674(33.21)	3043(38.72)	3910(50.84)	
No	20516(63.27)	6541(74.14)	5377(66.79)	4817(61.28)	3781(49.16)	
Diabetes(%)						< 0.001
Yes	4554(14.05)	1004(11.38)	1098(13.64)	1078(13.72)	1374(17.87)	
No	27870(85.95)	7818(88.62)	6953(86.36)	6782(86.28)	6317(82.13)	
CHF(%)						< 0.001
Yes	1078(3.32)	146(1.65)	191(2.37)	231(2.94)	510(6.63)	
No	31346(96.68)	8676(98.35)	7860(97.63)	7629(97.06)	7181(93.37)	
CHD(%)						< 0.001
Yes	2428(7.49)	404(4.58)	528(6.56)	636(8.09)	860(11.18)	
No	29996(92.51)	8418(95.42)	7523(93.44)	7224(91.91)	6831(88.82)	
Stroke(%)						< 0.001
Yes	1246(3.84)	261(2.96)	271(3.37)	300(3.82)	414(5.38)	
No	31178(96.16)	8561(97.04)	7780(96.63)	7560(96.18)	7277(94.62)	
Lung disease (%)						0.808
Yes	2349(7.24)	622(7.05)	603(7.49)	542(6.90)	582(7.57)	
No	30075(92.76)	8200(92.95)	7448(92.51)	7318(93.10)	7109(92.43)	
Liver disease(%)						< 0.001
Yes	1349(4.16)	281(3.19)	316(3.92)	358(4.55)	394(5.12)	
No	31075(95.84)	8541(96.81)	7735(96.08)	7502(95.45)	7297(94.88)	
Thyroid disease (%)						< 0.001
Yes	3403(10.50)	1050(11.90)	844(10.48)	778(9.90)	731(9.50)	
No	29021(89.50)	7772(88.10)	7207(89.52)	7082(90.10)	6960(90.50)	
Cancer(%)						< 0.001
Yes	3128(9.65)	741(8.40)	785(9.75)	774(9.85)	828(10.77)	
No	29296(90.35)	8081(91.60)	7266(90.25)	7086(90.15)	6863(89.23)	
Depressive symptoms(%)						< 0.001
Yes	3421(10.55)	1034(11.72)	854(10.61)	759(9.66)	774(10.06)	
No	29,003(89.45)	7788(88.28)	7197(89.39)	7101(90.34)	6917(89.94)	
PIR(%)	2.54(1.63)	2.50(1.63)	2.56(1.63)	2.56(1.64)	2.55(1.61)	0.033
Body mass index, kg/m^2^	29.27(6.98)	27.02(6.15)	28.87(6.69)	29.93(6.83)	31.60(7.45)	< 0.001
Waist Circumference (cm)	99.58(16.37)	92.56(14.77)	98.19(15.49)	101.91(15.56)	106.70(16.32)	< 0.001
WBC (1000 cells/uL)	7.24(3.30)	7.02(2.17)	7.21(2.56)	7.27(2.35)	7.51(5.28)	< 0.001
LYM (1000 cells/uL)	2.18(2.37)	2.15(1.03)	2.19(1.59)	2.19(1.19)	2.23(4.30)	0.036
RBC (million cells/uL)	4.68(0.50)	4.50(0.43)	4.66(0.48)	4.78(0.49)	4.80(0.55)	< 0.001
Hemoglobin (g/dL)	14.09(1.54)	13.50(1.40)	14.05(1.46)	14.42(1.47)	14.49(1.61)	< 0.001
Hematocrit(%)	41.59(4.26)	39.95(3.88)	41.47(4.06)	42.49(4.09)	42.68(4.45)	< 0.001
RDW (%)	13.33(1.38)	13.36(1.56)	13.28(1.31)	13.27(1.23)	13.39(1.36)	0.306
PLT(1000 cells/uL)	247.38(66.39)	253.56(67.44)	250.13(66.81)	243.48(65.76)	241.39(64.59)	< 0.001
Albumin(g/L)	42.27(3.38)	41.90(3.24)	42.27(3.33)	42.52(3.36)	42.44(3.57)	< 0.001
ALT(U/L)	21[16,28]	18[14,23]	20[16,27]	22[17,30]	24[18,34]	< 0.001
AST(U/L)	23[19,27]	21[18,25]	22[19,27]	23[20,28]	25[21,30]	< 0.001
BUN(mmol/L)	4.94(2.15)	4.34(1.61)	4.73(1.77)	4.98(1.94)	5.79(2.87)	< 0.001
Total-Chol(mmol/L)	5.00(1.09)	4.92(1.04)	5.00(1.10)	5.02(1.08)	5.05(1.13)	< 0.001
Creatinine(µmol/L)	76.02[63.65,89.28]	64.53[55.69,75.14]	72.49[63.65,84.86]	80.44[69.84,91.94]	88.40[77.35,104.31]	< 0.001
Glucose(mmol/L)	5.75(2.23)	5.70(2.67)	5.70(2.14)	5.72(1.99)	5.89(1.97)	< 0.001
Triglycerides(mmol/L)	1.37[0.91,2.11]	1.13[0.77,1.72]	1.31[0.89,2.01]	1.47[0.99,2.24]	1.65[1.12,2.51]	< 0.001
Uric acid (µmol/L)	326.56(85.82)	228.25(31.71)	297.80(15.38)	351.96(16.86)	443.46(55.49)	< 0.001
HDL- C(mmol/L)	1.37(0.42)	1.51(0.43)	1.39(0.41)	1.31(0.39)	1.24(0.39)	< 0.001

Q1: ≤267.7µmol/L; Q2:267.8–327.0µmol/L ; Q3:327.1-380.7µmol/L; Q4:≥380.8µmol/L.

Mean ± SD for continuous variables: P value was calculated by weighted ANOVA test. % for categorical variables: P value was calculated by weighted chi-square test. Median [interquartile range] for continuous variables: P value was calculated by weighted Kruskal-Wallis H test.

PIR, Family poverty income ratio; CHD, Coronary heart disease, CHF, Congestive heart failure; LYM, Lymphocyte number; RDW, Red cell distribution width; PLT, Platelet count ; ALT, Alanine Aminotransferase; AST, Aspartate Aminotransferase; BUN, Blood Urea Nitrogen; HDL-C, high-density lipoprotein cholesterol.

The characteristics of participants with and without depressive symptoms are summarized in Supplementary Table 1. No statistically significant differences were observed between the two groups in terms of age, ethnicity/ethnicity, alcohol use, and total cholesterol levels (all *P* > 0.05), as detailed in Supplementary Table 1. However, among participants with depressive symptoms, a higher proportion of females, individuals with education levels ≤ 11th Grade, those who were widowed/divorced/separated, and smokers were noted (all *P* < 0.05). Additionally, statistically significant differences were found between participants with and without depressive symptoms in terms of family poverty income ratio, BMI, waist circumference, and laboratory test results. Specifically, the uric acid levels in participants with depressive symptoms were significantly lower compared to those without depressive symptoms (320.93 ± 88.81 vs. 327.22 ± 85.44, *P* < 0.005).

### Association between UA and depressive symptoms

Because the effect value is not apparent, UA/100 is used to amplify the effect value by 100 times. After adjusting for age and gender, higher levels of UA/100 were found to be unrelated to the prevalence of depressive symptoms (*P* = 0.077). Subsequently, in Model 3 with adequate adjustment for covariates, a significant inverse association between elevated UA levels and the prevalence of depressive symptoms was observed (*P* < 0.001), Table 2. When UA was analyzed as a categorical variable, overall, compared to the first quartile, individuals with higher UA levels experienced a reduction in the risk of depressive symptoms by 9% (OR:0.910, 95% CI:0.797–1.040), 14.6%(OR:0.854, 95%CI:0.741–0.983), and 20.5% (OR:0.795, 95% CI:0.680–0.930), respectively, after full adjustment for covariates, Table 2. There was a significant decreasing trend in the prevalence of depressive symptoms with increasing UA levels (*P* for trend < 0.001), Table 2.

**Table 2:** Weighted logistic regression analysis of uric acid and depressive symptoms.

Uric acid	Model 1		Model 2		Model 3	
	OR(95%CI)	*P*	OR(95%CI)	*P*	OR(95%CI)	*P*
Continuous	0.916(0.861–0.974)	0.005	1.065(0.993–1.141)	0.077	0.876(0.819–0.938)	< 0.001
Q1	Ref.		Ref.		Ref.	
Q2	0.907(0.803–1.024)	0.116	1.039(0.917–1.178)	0.544	0.910(0.797–1.040)	0.166
Q3	0.855(0.747–0.978)	0.023	1.110(0.963–1.281)	0.150	0.854(0.741–0.983)	0.028
Q4	0.843(0.741–0.959)	0.009	1.189(1.020–1.386)	0.027	0.795(0.680–0.930)	0.004
***P*** for trend		< 0.001		0.039		< 0.001

### Smooth curve fitting, threshold effect analyses between UA and depressive symptoms

**Figure 2:**
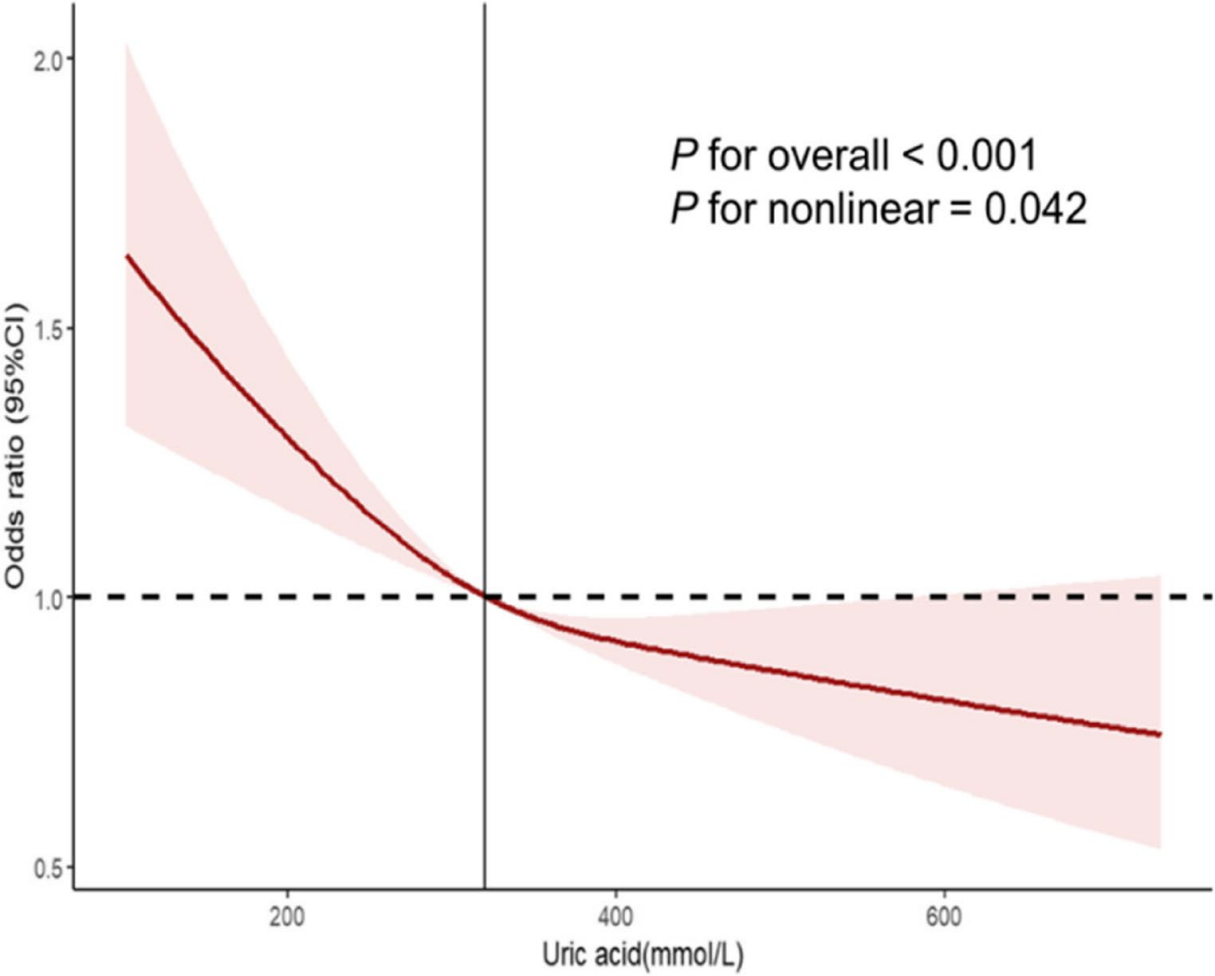
Restricted cubic spline analysis of the relationship between uric acid and depressive symptoms.

To further explore the relationship between UA and depressive symptoms, we conducted a threshold effect analysis using smooth curve fitting (Fig. 2). We identified a nonlinear association between UA and depressive symptoms, with an inflection point at 319.72µmol/L. When UA levels are below 319.72µmol/L, for every 100µmol/L increase in UA, the risk of depressive symptoms decreases by 21.7% (OR:0.783, 95%CI:0.668–0.918, *P* = 0.004). However, when UA levels exceed 319.72µmol/L, for every 100µmol/L increase, the risk of depressive symptoms decreases by 2% (OR:0.980; 95%CI: 0.972–0.988; *P* = 0.029) (Table 3).

**Table 3:** Analysis of the threshold effect of uric acid on depressive symptoms by two-piece linear regression model.

Inflection point	OR(95%CI)	*P*
≤ 319.72	0.783(0.668–0.918)	0.004
> 319.72	0.980(0.972–0.988)	0.029
Log likelihood ratio tests		< 0.001

### Subgroup analyses

**Figure 3:**
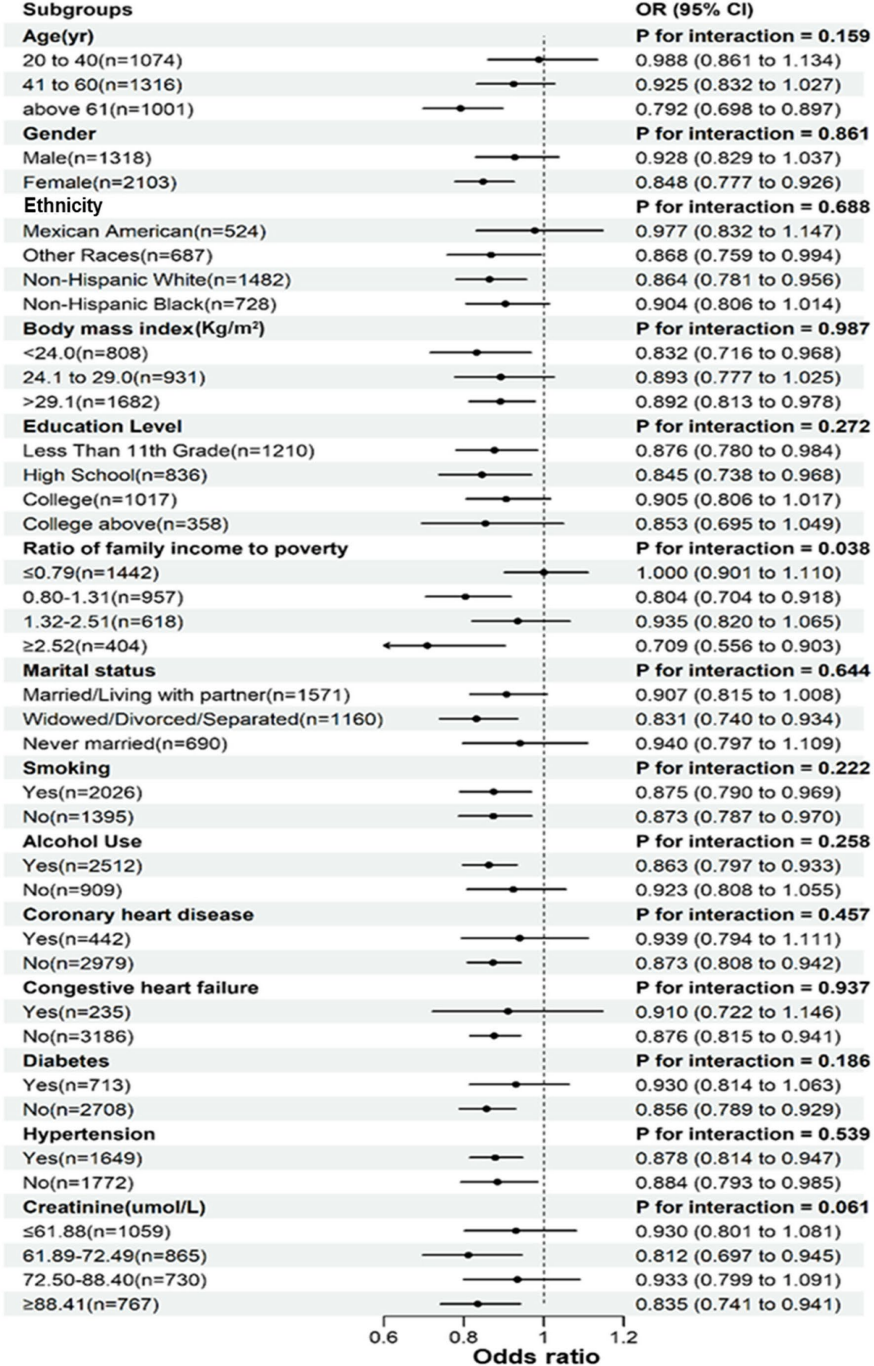
Subgroup weighted multivariate logistic regression analysis of forest plot.

To evaluate the stability of the correlation between UA and depressive symptoms across different subgroups, we conducted a subgroup analysis. The interaction test showed that there was no significant interaction between UA and depressive symptoms across different subgroups except for family poverty income ratio (all *P* > 0.05) (Fig. 3). This indicated that factors such as age (20–40/41–60/≥61 years), gender (male/female), ethnicity (Mexican Americans/other races/non-Hispanic whites/non-Hispanic blacks), education level (below 11th grade/high school or equivalent/college or AA degree/bachelor’s degree or above), BMI (< 24.0/ 24.1 to 29.0 />29.1), marital status (married/living with partner, widowed/divorced/separated, never married), smoking (yes/no), alcohol use (yes/no), diabetes (yes/no), coronary heart disease (yes/no), congestive heart failure (yes/no), hypertension (yes/no), and serum creatinine (≤ 61.88, 61.89–72.49, 72.50–88.40, ≥ 88.41) did not have a significant impact on the association between UA and depressive symptoms (all *P* > 0.05 for interactions). This suggested that the association between UA and the risk of depressive symptoms remained consistent across all subgroups, demonstrating a high level of stability and reliability.

## Discussion

The primary objective of this study was to evaluate the correlation between serum uric acid (UA) levels and depressive symptoms among US adults. This cross-sectional study utilized data from seven consecutive cycles of the National Health and Nutrition Examination Survey (NHANES) spanning from 2005 to 2018, comprising 32,424 participants. The results revealed a significant association between serum uric acid levels and depressive symptoms. Through detailed subgroup analyses and interaction testing, we further explored and confirmed this finding, elucidating a similar trend in their association. Further restrictive cubic spline analysis indicated a nonlinear relationship between UA and depressive symptoms. Specifically, When UA levels are below 319.72µmol/L, for every 100µmol/L increase in UA, the risk of depressive symptoms decreases by 21.7%. However, when UA levels exceed 319.72µmol/L, for every 100µmol/L increase, the risk of depressive symptoms decreases by 2%. These findings not only validate our initial hypothesis but also deepen our understanding of the complex interplay between serum uric acid and depressive symptoms among US adults, providing valuable clues and insights for further understanding the pathogenesis of adult depression.

Our study demonstrates a similar trend to previous reports. Data from the China Health and Retirement Longitudinal Study (CHARLS) involving 4845 female participants revealed a positive correlation between increasing serum uric acid levels and alleviation of depressive symptoms^26^. Similarly, another study conducted in Korea found that elderly women with lower uric acid levels had a relatively higher incidence of depressive symptoms, whereas this association was not significant in elderly men^28^. Our findings further corroborate this viewpoint. Through multifactorial logistic regression analysis of different gender and age subgroups, we observed a more significant negative correlation between uric acid and depressive symptoms in females compared to males, with this negative correlation being more pronounced in the elderly population aged 60 and above. Additionally, studies have indicated that among 1543 patients with depression, their uric acid levels were generally lower than those in the normal control group^31^. Another study involving 707 participants revealed a nonlinear relationship between lower uric acid levels upon admission and post-stroke depression upon discharge, identifying a threshold value of 300µmol/L^32^. While this study’s focus was limited to post-stroke patients and may not be generalizable to the general population, its findings intersect with ours. We utilized a representative sample of NHANE participants and found a nonlinear association between serum uric acid and depressive symptoms among US adults, with a threshold value of 319.72µmol/L. These collective findings provide valuable insights into understanding the relationship between serum uric acid and depressive symptoms.

In our study, based on multivariable logistic regression analysis and restrictive cubic spline plots, a significant negative correlation was observed between serum uric acid levels and the risk of depressive symptoms in American adults. The potential mechanisms underlying the negative correlation between serum uric acid (UA) levels and the risk of adult depressive symptoms encompass several aspects. Firstly, UA possesses antioxidant properties as a potent endogenous antioxidant capable of scavenging free radicals and reactive oxygen species (ROS) to maintain cellular health^33^. Given the close association between depression and oxidative stress, an imbalance between ROS production and antioxidant defense mechanisms can lead to cellular damage and dysfunction. Therefore, elevated levels of UA may mitigate oxidative stress, thus preventing neuronal damage associated with depression. Secondly, UA exhibits neuroprotective effects, as evidenced by preclinical studies demonstrating its ability to reduce neuro-inflammation and prevent neuronal apoptosis^34^, thereby preserving the integrity and function of the nervous system. By maintaining the health of neurons, high levels of UA may enhance resilience against the development of depressive symptoms. Additionally, UA regulates neurotransmitter systems, including dopamine and glutamate, which are implicated in depression^35^. Dopamine dysregulation is a common feature of depression, and UA has been shown to enhance dopamine release and receptor function, thereby improving mood. Moreover, UA may also modulate glutamate neurotransmission, which plays a crucial role in mood regulation and is closely related to the pathophysiology of depression^36^. Furthermore, UA exhibits anti-inflammatory effects, which are pertinent as chronic low-grade inflammation is a key factor in depression pathogenesis. As an anti-inflammatory agent, UA inhibits the production of pro-inflammatory cytokines, thereby alleviating inflammation associated with depression^37^. Additionally, UA is involved in regulating endothelial function and cerebral blood flow, both of which are disrupted in depressed patients^38^. By improving endothelial function and promoting cerebral blood flow, UA may reduce the risk of depressive symptoms. Lastly, UA interacts with other biological systems involved in regulating mood and stress responses^39^, such as the renin-angiotensin system and purinergic system. Through modulation of these systems, UA may indirectly impact depressive symptoms. In summary, UA is negatively correlated with the risk of adult depressive symptoms through multiple mechanisms including antioxidant activity, neuroprotection, neurotransmitter regulation, anti-inflammatory effects, and modulation of endothelial function and cerebral blood flow. These findings provide important insights for understanding the pathogenesis of depression and exploring novel treatment strategies.

Furthermore, an interesting finding of our study is that although serum uric acid levels are negatively correlated with the risk of depressive symptoms in American adults, as demonstrated by restrictive cubic spline analysis, this negative correlation exhibits nonlinearity with a inflection point at 319.72 µmol/L. When UA levels are below 319.72 µmol/L, an increase of 100 µmol/L in UA can decrease the risk of depression by 21.7%. However, once UA exceeds 319.72 µmol/L, each 100 µmol/L increase in UA only reduces the risk of depression by 2%. This study finding provides important evidence for clinicians in assessing the risk of depression in patients. Specifically, when serum uric acid (UA) levels are below 319.72 µmol/L, individuals may face a higher risk of depression; conversely, when UA levels exceed this threshold, the risk of depression may be relatively lower for individuals. Therefore, serum UA levels hold promise as a biomarker for assessing the risk of depression, providing robust support for the diagnostic process. Clinicians can incorporate UA measurements into routine assessment protocols to more accurately identify individuals at risk of depression. Furthermore, a comprehensive understanding of the association between UA levels and the risk of depression is crucial for developing effective treatment strategies. Interventions aimed at increasing UA levels, such as dietary adjustments or pharmacological treatments, may help reduce the risk of depression for individuals with lower UA levels. These interventions not only contribute to improving overall health but may also have a positive impact on the prevention and treatment of depression.

In addition, our analysis suggests that the specific reasons for the inconsistent risk of depression in American adults with serum uric acid levels below or above 319.72 µmol/L may be related to the following factors as the serum uric acid level increases. When UA levels are below 319.72 µmol/L, several physiological processes may contribute to an increased risk of depression. Firstly, weakened antioxidant activity may occur, indicating a decrease in the body’s ability to counteract oxidative stress. Given the close association between oxidative stress and the pathophysiology of depression^40^, elevating UA levels could potentially enhance antioxidant defenses, reduce oxidative damage, and consequently lower the risk of depression. Secondly, a decrease in neuroprotective effects may be observed, as lower UA levels may correlate with diminished neuroprotection, rendering neurons more susceptible to damage and functional impairment^41^. By increasing UA levels, activation of neuroprotective mechanisms could be achieved, maintaining neuronal integrity and reducing the risk of depression. Additionally, dysregulation of neurotransmitters may occur, as UA plays a crucial role in modulating neurotransmitter systems involved in mood regulation, such as dopamine and glutamate. Increasing UA levels may help restore neurotransmitter balance, thereby improving mood and reducing the risk of depression^42^. Furthermore, weakened anti-inflammatory properties may be observed, as lower UA levels may be associated with increased inflammation, which is closely linked to the pathogenesis of depression^43^. Elevating UA levels may exert anti-inflammatory effects, alleviating the inflammatory process and subsequently lowering the risk of depression. Lastly, impaired endothelial function and cerebral blood flow may occur, as lower UA levels may be associated with endothelial dysfunction and reduced cerebral blood flow, both of which are linked to depression. Increasing UA levels could improve endothelial function, increase cerebral blood flow, and consequently reduce the risk of depression^44^. However, when UA levels exceed 319.72 µmol/L, the situation may change. On one hand, the effectiveness of additional UA in reducing the risk of depression may gradually diminish, possibly due to saturation of protective mechanisms associated with UA. On the other hand, beyond a certain threshold, other factors such as genetic predisposition, environmental stressors, or comorbid medical conditions may have a greater impact on the risk of depression, overshadowing the potential benefits of further increasing UA levels. In summary, the impact of UA levels on the risk of depression is dual-sided and may depend on whether UA levels are below or above 319.72 µmol/L, highlighting the complexity of the relationship between UA and depression. Further research is needed to deeply understand the specific mechanisms underlying this phenomenon and to validate these findings in different populations.

## Limitations and strengths

The current study has several notable limitations. First, due to its cross-sectional design, establishing a definitive causal relationship between serum uric acid levels and depressive symptoms presents a major challenge. Further prospective studies are urgently needed to elucidate the potential link between these two factors and to provide stronger evidence. Second, the limitations of the existing database make it difficult to comprehensively include all dietary and environmental factors associated with uric acid levels, which may partially affect the comprehensiveness and accuracy of the study results. Additionally, although the PHQ-9, a self-report tool for assessing depressive symptoms, is widely accepted in clinical and epidemiological studies, with its high sensitivity and specificity well validated, this method still relies on subjective reporting, which may introduce bias. Moreover, individuals who are taking antidepressant medications but score normally on the PHQ-9 may be overlooked. Finally, this study focuses on adults aged 20 and older, so caution is advised when interpreting the findings for individuals under 20 years of age.

However, the current study also possesses several significant strengths. Firstly, we focus on serum uric acid levels as a practical and readily obtainable indicator, providing a robust tool for exploring its relationship with depressive symptoms. Secondly, through careful utilization of data from the National Health and Nutrition Examination Survey (NHANES), we ensured high data quality and broad generalizability of results. These data were obtained through meticulous multi-stage probability sampling design, representing the entire non-institutionalized civilian population of the United States. Thirdly, we rigorously controlled for various confounding factors, including sociodemographic characteristics, dietary energy intake, and various comorbidities, aiding in a more accurate assessment of the relationship between serum uric acid levels and depressive symptoms. Finally, this study revealed for the first time a nonlinear negative correlation between serum uric acid levels and the risk of depressive symptoms among American adults, calculating a specific inflection point. This finding holds important public health implications for preventing depressive episodes.

## Conclusion

In this comprehensive investigation, we observed a significant nonlinear negative correlation between serum uric acid levels and depressive symptoms among the adult population in the United States. Of particular note is the evident inflection point at a serum uric acid level of 319.72 µmol/L. This finding suggests a potentially significant role for uric acid in the identification and treatment of depression. However, further prospective research is warranted to precisely elucidate the causal relationship between elevated serum uric acid levels and the risk of depression, thereby necessitating validation and deeper exploration.

## Supplementary Information

Supplementary Material 1.

## Data Availability

Publicly available datasets were analyzed in this study. All the raw data used in this study are derived from the public NHANES data portal (https://wwwn.cdc.gov/nchs/nhanes/Default.aspx).

## References

[1] Lu, B., Lin, L. & Su, X. Global burden of depression or depressive symptoms in children and adolescents: a systematic review and meta-analysis. *J. Affect. Disord.* **354**, 553–562. 10.1016/j.jad.2024.03.074 (2024). 38490591 10.1016/j.jad.2024.03.074

[2] Li, Z. B. et al. Burden of depression in adolescents in the Western Pacific Region from 1990 to 2019: an age-period-cohort analysis of the Global Burden of Disease study. *Psychiatry Res.* **336**, 115889. 10.1016/j.psychres.2024.115889 (2024). 38621309 10.1016/j.psychres.2024.115889

[3] Liu, Q. et al. Changes in the global burden of depression from 1990 to 2017: findings from the Global Burden of Disease study. *J. Psychiatr Res.* **126**, 134–140. 10.1016/j.jpsychires.2019.08.002 (2020). 31439359 10.1016/j.jpsychires.2019.08.002

[4] Shorey, S., Ng, E. D. & Wong, C. H. J. Global prevalence of depression and elevated depressive symptoms among adolescents: a systematic review and meta-analysis. *Br. J. Clin. Psychol.* **61** (2), 287–305. 10.1111/bjc.12333 (2022). 34569066 10.1111/bjc.12333

[5] Daly, M., Sutin, A. R. & Robinson, E. Depression reported by US adults in 2017–2018 and March and April 2020. *J. Affect. Disord*. **278**, 131–135. 10.1016/j.jad.2020.09.065 (2021). 32956962 10.1016/j.jad.2020.09.065 PMC7490280

[6] Cuijpers, P. et al. Psychotherapy for subclinical depression: meta-analysis. *Br. J. Psychiatry*. **205** (4), 268–274. 10.1192/bjp.bp.113.138784 (2014). 25274315 10.1192/bjp.bp.113.138784 PMC4180844

[7] Li, G. H. et al. Evaluation of bi-directional causal association between depression and cardiovascular diseases: a mendelian randomization study. *Psychol. Med.* **52** (9), 1765–1776. 10.1017/S0033291720003566 (2022). 33032663 10.1017/S0033291720003566

[8] Nong, Y., Wu, G., Lu, J., Wei, X. & Yu, D. The mediating role of obesity in the development of depression in individuals with diabetes: a population-based study from NHANES 2005–2014. *J. Affect. Disord*. **351**, 977–982. 10.1016/j.jad.2024.02.036 (2024). 38355056 10.1016/j.jad.2024.02.036

[9] O’Connor, E. A. et al. Depression and suicide risk screening: updated evidence report and systematic review for the US Preventive Services Task Force. *JAMA*. **329** (23), 2068–2085. 10.1001/jama.2023.7787 (2023). 37338873 10.1001/jama.2023.7787 PMC13533462

[10] Zhang, G. et al. The association between serum albumin and depressive symptoms: a cross-sectional study of NHANES data during 2005–2018. *BMC Psychiatry*. **23** (1), 448. 10.1186/s12888-023-04935-1 (2023). 37340352 10.1186/s12888-023-04935-1 PMC10283330

[11] Wen, S., Arakawa, H. & Tamai, I. Uric acid in health and disease: from physiological functions to pathogenic mechanisms. *Pharmacol. Ther.* **256**, 108615. 10.1016/j.pharmthera.2024.108615 (2024). 38382882 10.1016/j.pharmthera.2024.108615

[12] Casanova, A. G., Morales, A. I., Vicente-Vicente, L. & López-Hernández, F. J. Effect of uric acid reduction on chronic kidney disease. Systematic review and meta-analysis. *Front. Pharmacol.* **15**, 1373258. 10.3389/fphar.2024.1373258 (2024). 38601468 10.3389/fphar.2024.1373258 PMC11005459

[13] Wium-Andersen, M. K., Kobylecki, C. J., Afzal, S. & Nordestgaard, B. G. Association between the antioxidant uric acid and depression and antidepressant medication use in 96 989 individuals. *Acta Psychiatr Scand.* **136** (4), 424–433. 10.1111/acps.12793 (2017). 28845530 10.1111/acps.12793

[14] Chen, T. S. et al. Investigating the nexus of metabolic syndrome, serum uric acid, and dementia risk: a prospective cohort study. *BMC Med.* **22** (1), 115. 10.1186/s12916-024-03302-5 (2024). 38481272 10.1186/s12916-024-03302-5 PMC10938845

[15] Mohammadi, M. et al. Uric acid and glaucoma: a systematic review and meta-analysis. *Front. Med. (Lausanne)*. **10**, 1159316. 10.3389/fmed.2023.1159316 (2023). 37575992 10.3389/fmed.2023.1159316 PMC10422028

[16] Koros, C. et al. Serum uric acid as a putative biomarker in Prodromal Parkinson’s Disease: longitudinal data from the PPMI Study. *J. Parkinsons Dis.* **13** (5), 811–818. 10.3233/JPD-230007 (2023). 37424476 10.3233/JPD-230007 PMC10473106

[17] Geng, R. et al. Elevated serum uric acid is associated with cognitive improvement in older American adults: a large, population-based-analysis of the NHANES database. *Front. Aging Neurosci.* **14**, 1024415. 10.3389/fnagi.2022.1024415 (2022). 36570535 10.3389/fnagi.2022.1024415 PMC9772611

[18] Fabbrini, E., Serafini, M., Colic Baric, I., Hazen, S. L. & Klein, S. Effect of plasma uric acid on antioxidant capacity, oxidative stress, and insulin sensitivity in obese subjects. *Diabetes*. **63** (3), 976–981. 10.2337/db13-1396 (2014). 24353177 10.2337/db13-1396 PMC3931399

[19] Yang, L. et al. Whole blood cadmium levels and depressive symptoms in Chinese young adults: a prospective cohort study combing metabolomics. *J. Hazard. Mater.* **465**, 132968. 10.1016/j.jhazmat.2023.132968 (2024). 38000288 10.1016/j.jhazmat.2023.132968

[20] Romaszko, J. et al. Are the levels of uric acid associated with biometeorological conditions? *Sci. Total Environ.* **819**, 152020. 10.1016/j.scitotenv.2021.152020 (2022). 35007576 10.1016/j.scitotenv.2021.152020

[21] Ba DM, Gao X, Al-Shaar L, Muscat JE, Chinchilli VM, Beelman RB, Richie JP. Mushroom intake and depression: A population-based study using data from the US National Health and Nutrition Examination Survey (NHANES), 2005-2016.* J. Affect. Disord*. **294**, 686–692. 10.1016/j.jad.2021.07.080 (2021). 10.1016/j.jad.2021.07.080 34333177

[22] Liu X, Liu X, Wang Y, Zeng B, Zhu B, Dai F. Association between depression and oxidative balance score: National Health and Nutrition Examination Survey (NHANES) 2005-2018. *J. Affect. Disord*. **337**, 57–65. 10.1016/j.jad.2023.05.071 (2023). 10.1016/j.jad.2023.05.071 37244542

[23] Hu PW, Zhang XL, Yan XT, Qi C, Jiang GJ. Association between depression and endometriosis using data from NHANES 2005-2006. *Sci. Rep*. **13** (1), 18708. 10.1038/s41598-023-46005-2 (2023). 10.1038/s41598-023-46005-2 PMC10618216 37907559

[24] Kroenke K, Spitzer RL, Williams JB. The PHQ-9: validity of a brief depression severity measure. *J. Gen. Intern. Med*. **16** (9), 606–13. 10.1046/j.1525-1497.2001.016009606.x (2001). 10.1046/j.1525-1497.2001.016009606.x PMC1495268 11556941

[25] Xiao Y, Huang W. Association of Dietary Inflammatory Index With Depression and Suicidal Ideation in Older Adult: Results From the National Health and Nutrition Examination Surveys 2005-2018. *Front Psychiatry*. **13**, 944154. 10.3389/fpsyt.2022.944154 (2022). 10.3389/fpsyt.2022.944154 PMC9294216 35865298

[26] Chen, J., Zhou, W. & Huang, Y. Association between serum uric acid levels and depressive symptoms according to menopausal status. *J. Affect. Disord*. **350**, 240–246. 10.1016/j.jad.2024.01.108 (2024). 38220113 10.1016/j.jad.2024.01.108

[27] Rhee, S. J., Lee, H. & Ahn, Y. M. Association between serum uric acid and depressive symptoms stratified by low-grade inflammation status. *Sci. Rep.* **11** (1), 20405. 10.1038/s41598-021-99312-x (2021). 34650110 10.1038/s41598-021-99312-x PMC8516956

[28] Kim, J. O., Park, G. N., Oh, J. W. & Lee, S. Association between uric acid and depressive symptoms in older adults: the Korea National Health and Nutrition Examination Survey. *Int. J. Geriatr. Psychiatry*. **38** (7), e5963. 10.1002/gps.5963 (2023). 37395133 10.1002/gps.5963

[29] Zhang, C. et al. Association between eicosapentaenoic acid use and the risk of depressive symptoms in US adults: analyses from NHANES 2005–2018. *J. Affect. Disord*. **354**, 62–67. 10.1016/j.jad.2024.03.055 (2024). 38479498 10.1016/j.jad.2024.03.055

[30] Harshfield, E. L. et al. Emerging risk factors collaboration. Association between depressive symptoms and Incident Cardiovascular diseases. *JAMA*. **324** (23), 2396–2405. 10.1001/jama.2020.23068 (2020). 33320224 10.1001/jama.2020.23068 PMC7739139

[31] Meng, X., Huang, X., Deng, W., Li, J. & Li, T. Serum uric acid a depression biomarker. *PLoS One*. **15** (3), e0229626. 10.1371/journal.pone.0229626 (2020). 32130258 10.1371/journal.pone.0229626 PMC7055893

[32] Li, G. et al. Lower serum uric acid is Associated with post-stroke depression at discharge. *Front. Psychiatry*. **11**, 52. 10.3389/fpsyt.2020.00052 (2020). 32132938 10.3389/fpsyt.2020.00052 PMC7040095

[33] Ryan, E. M. et al. Antioxidant properties of citric acid interfere with the uricase-based measurement of circulating uric acid. *J. Pharm. Biomed. Anal.* **164**, 460–466. 10.1016/j.jpba.2018.11.011 (2019). 30447534 10.1016/j.jpba.2018.11.011 PMC6298802

[34] Otani, N., Hoshiyama, E., Ouchi, M., Takekawa, H. & Suzuki, K. Uric acid and neurological disease: a narrative review. *Front. Neurol.* **14**, 1164756. 10.3389/fneur.2023.1164756 (2023). 37333005 10.3389/fneur.2023.1164756 PMC10268604

[35] Tang, Y., Du, Y., Ye, J., Deng, L. & Cui, W. Intestine-targeted explosive hydrogel Microsphere promotes uric acid excretion for gout therapy. *Adv. Mater.* **36** (3), e2310492. 10.1002/adma.202310492 (2024). 37997010 10.1002/adma.202310492

[36] Zhong, R. et al. A cross-sectional study on the association of serum uric acid levels with depressive and anxiety symptoms in people with epilepsy. *BMC Psychiatry* **21**(1), 17. 10.1186/s12888-020-03019-8 (2021) (PMID: 33413258). 33413258 10.1186/s12888-020-03019-8 PMC7791969

[37] Liu, J. et al. Co-delivery of indomethacin and uricase as a new strategy for inflammatory diseases associated with high uric acid. *Drug Deliv Transl Res. *10.1007/s13346-023-01487-5 (2023). 10.1007/s13346-023-01487-5 38127247

[38] De Becker, B. & Van De Borne, P. Serum uric acid: a futile bystander in endothelial function? *Blood Press.* **32** (1), 2237123. 10.1080/08037051.2023.2237123 (2023). 37470459 10.1080/08037051.2023.2237123

[39] Hwang, J. S., Yeo, J. N. & Hwang, I. C. Serum uric acid levels in people with anxiety: a Korean nationwide survey. *J. Affect. Disord*. **352**, 138–139. 10.1016/j.jad.2024.02.058 (2024). 38367496 10.1016/j.jad.2024.02.058

[40] Rossato, B. G. & Moresco, R. N. Effects of pH on the in vitro antioxidant activity of Uric Acid. *Clin. Lab.* **70** (3). 10.7754/Clin.Lab.2023.230903 (2024). 10.7754/Clin.Lab.2023.230903 38469767

[41] Noor, R. I. I. et al. Serum uric acid and serum lipid levels in patients with Acute ischemic stroke admitted in Mymensingh Medical College Hospital. *Mymensingh Med. J.* **33** (2), 402–410 (2024). 38557518

[42] Salimi, A., Mamkhezri, H. & Hallaj, R. Simultaneous determination of ascorbic acid, uric acid and neurotransmitters with a carbon ceramic electrode prepared by sol-gel technique. *Talanta*. **70** (4), 823–832. 10.1016/j.talanta.2006.02.015 (2006). 18970846 10.1016/j.talanta.2006.02.015

[43] Xie, F. et al. Association between systemic immune-inflammation index and serum uric acid in U.S. adolescents: a population-based study. *Nutr. Metab. Cardiovasc. Dis.* **34** (1), 206–213. 10.1016/j.numecd.2023.10.008 (2024). 37996371 10.1016/j.numecd.2023.10.008

[44] Tang, J. et al. Association between serum uric acid and impaired endothelial function: the circulatory risk in communities Study. *J. Atheroscler Thromb.* **29** (10), 1534–1546. 10.5551/jat.63199 (2022). 34853212 10.5551/jat.63199 PMC9529376

