# Supplementary material for: Association between uric acid and the risk of depressive symptoms in US adults: results from NHANES 2005–2018
Source: Sci Rep. 2024 Oct 15;14:24097. doi: 10.1038/s41598-024-74869-5 (PMC11480327; doi:10.1038/s41598-024-74869-5)
Supplement: Supplementary file 1 — Supplementary Material 1. [file 41598_2024_74869_MOESM1_ESM.docx]

Supplementary Table 1. Characteristics of the participants by depressive symptoms.

| Variables | Overall | Non-Depression | Depression | P |
| --- | --- | --- | --- | --- |
|  | 32424 | n=29003 | n=3421 |  |
| Age(mean(SD)) | 50.18(17.69) | 50.18(17.81) | 50.12(16.58) | 0.238 |
| Gender(%) |  |  |  | <0.001 |
| Male | 16178(49.90) | 14860(51.24) | 1318(38.53) |  |
| Female | 16246(50.10) | 14143(48.76) | 2103(61.47) |  |
| Ethnicity (%) |  |  |  | 0.918 |
| Mexican American | 5072(15.64) | 4548(15.68) | 524(15.32) |  |
| Other Hispanic | 6508(20.07) | 5821(20.07) | 687(20.08) |  |
| Non-Hispanic White | 14062(43.37) | 12580(43.37) | 1482(43.32) |  |
| Non-Hispanic Black | 6782(20.92) | 6054(20.87) | 728(21.28) |  |
| Education Level (%) |  |  |  | <0.001 |
| ≤11th Grade | 7881(24.31) | 6671(23.00) | 1210(35.37) |  |
| High School | 7483(23.08) | 6647(22.92) | 836(24.44) |  |
| College | 9622(29.68) | 8605(29.67) | 1017(29.73) |  |
| College above | 7438(22.94) | 7080(24.41) | 358(10.46) |  |
| Marital status (%) |  |  |  | <0.001 |
| Married/Living with partner | 19327(59.61) | 17756(61.22) | 1571(45.92) |  |
| Widowed/Divorced/Separated | 7335(22.62) | 6175(21.29) | 1160(33.91) |  |
| Never married | 5762(17.77) | 5072(17.49) | 690(20.17) |  |
| Smoking (%) |  |  |  | <0.001 |
| Yes | 14726(45.42) | 12700(43.79) | 2026(59.22) |  |
| No | 17698(54.58) | 16303(56.21) | 1395(40.78) |  |
| Alcohol use (%) |  |  |  | 0.355 |
| Yes | 23763(73.29) | 21251(73.27) | 2512(73.43) |  |
| No | 8661(26.71) | 7752(26.73) | 909(26.57) |  |
| Hypertension(%) |  |  |  | <0.001 |
| Yes | 11908(36.73) | 10259(35.37) | 1649(48.20) |  |
| No | 20516(63.27) | 18744(64.63) | 1772(51.80) |  |
| Diabetes(%) |  |  |  | <0.001 |
| Yes | 4554(14.05) | 3841(13.24) | 713(20.84) |  |
| No | 27870(85.95) | 25162(86.76) | 2708(79.16) |  |
| Congestive heart failure (%) |  |  |  | <0.001 |
| Yes | 1078(3.32) | 843(2.91) | 235(6.87) |  |
| No | 31346(96.68) | 28160(97.09) | 3186(93.13) |  |
| Coronary heart disease (%) |  |  |  | <0.001 |
| Yes | 2428(7.49) | 1986(6.85) | 442(12.92) |  |
| No | 29996(92.51) | 27017(93.15) | 2979(87.08) |  |
| Stroke(%) |  |  |  | <0.001 |
| Yes | 1246(3.84) | 982(3.39) | 264(7.72) |  |
| No | 31178(96.16) | 28021(96.61) | 3157(92.28) |  |
| Lung disease (%) |  |  |  | <0.001 |
| Yes | 2349(7.24) | 1775(6.12) | 574(16.78) |  |
| No | 30075(92.76) | 27228(93.88) | 2847(83.22) |  |
| Liver disease(%) |  |  |  | <0.001 |
| Yes | 1349(4.16) | 1045(3.60) | 304(8.89) |  |
| No | 31075(95.84) | 27958(96.40) | 3117(91.11) |  |
| Thyroid disease (%) |  |  |  | <0.001 |
| Yes | 3403(10.50) | 2865(9.88) | 538(15.73) |  |
| No | 29021(89.50) | 26138(90.12) | 2883(84.27) |  |
| Cancer(%) |  |  |  | <0.001 |
| Yes | 3128(9.65) | 2735(9.43) | 393(11.49) |  |
| No | 29296(90.35) | 26268(90.57) | 3028(88.51) |  |
| Family poverty income ratio,% | 2.54(1.63) | 2.63(1.63) | 1.82(1.43) | <0.001 |
| Body mass index, kg/m^2^ | 29.27(6.98) | 29.07(6.79) | 30.98(8.23) | <0.001 |
| Waist Circumference (cm) | 99.58(16.37) | 99.18(16.13) | 102.93(17.92) | <0.001 |
| White blood cell count(1000 cells/uL) | 7.24(3.30) | 7.19(3.35) | 7.73(2.76) | <0.001 |
| Lymphocyte number (1000 cells/uL) | 2.18(2.37) | 2.17(2.45) | 2.29(1.54) | 0.001 |
| Red blood cell count (million cells/uL) | 4.68(0.50) | 4.69(0.50) | 4.61(0.51) | <0.001 |
| Hemoglobin (g/dL) | 14.09(1.54) | 14.12(1.53) | 13.87(1.60) | <0.001 |
| Hematocrit(%) | 41.59(4.26) | 41.66(4.24) | 40.97(4.42) | <0.001 |
| Red cell distribution width (%) | 13.33(1.38) | 13.30(1.35) | 13.59(1.58) | <0.001 |
| Platelet count (1000 cells/uL) | 247.38(66.39) | 246.40(65.67) | 255.71(71.67) | <0.001 |
| Albumin(g/L) | 42.27(3.38) | 42.36(3.34) | 41.48(3.59) | <0.001 |
| Alanine Aminotransferase (U/L) | 21[16,28] | 21[16,28] | 20[15,28] | 0.015 |
| Aspartate Aminotransferase (U/L) | 23[19,27] | 23[19,27] | 22[19,27] | 0.001 |
| Blood Urea Nitrogen (mmol/L) | 4.94(2.15) | 4.95(2.11) | 4.82(2.44) | 0.001 |
| Total-Chol(mmol/L) | 5.00(1.09) | 4.99(1.08) | 5.03(1.17) | 0.294 |
| Creatinine(µmol/L) | 76.02[63.65,89.28] | 76.02[63.65,90.17] | 72.49[61.88,88.40] | <0.001 |
| Glucose(mmol/L) | 5.75(2.23) | 5.71(2.13) | 6.09(2.87) | <0.001 |
| Triglycerides(mmol/L) | 1.37[0.91,2.11] | 1.36[0.90,2.09] | 1.52[0.98,2.30] | <0.001 |
| Uric acid (µmol/L) | 326.56(85.82) | 327.22(85.44) | 320.93(88.81) | 0.005 |
| HDL- C(mmol/L) | 1.37(0.42) | 1.37(0.42) | 1.33(0.41) | <0.001 |

Mean ± SD for continuous variables: P value was calculated by weighted ANOVA test. % for categorical variables: P value was calculated by weighted chi-square test. Median [interquartile range] for continuous variables: P value was calculated by weighted Kruskal-Wallis H test.
